# Real-time gas mass spectroscopy by multivariate analysis

**DOI:** 10.1038/s41598-023-33188-x

**Published:** 2023-04-13

**Authors:** Leonardo Franceschelli, Carla Ciricugno, Maurizio Di Lorenzo, Aldo Romani, Annachiara Berardinelli, Marco Tartagni, Raffaele Correale

**Affiliations:** 1 Department of Electrical, Electronic and Information Engineering (DEI), Alma Mater Studiorum University of Bologna (IT), Bologna, Italy; 2 NanoTech Analysis S.R.L., Torino, Italy; 3 Department of Industrial Engineering, University of Trento (IT), Trento, Italy; 4 C3A Center Agriculture Food Environment, University of Trento (IT), Trento, Italy

**Keywords:** Engineering, Mathematics and computing, Nanoscience and technology, Physics

## Abstract

Early and significant results for a real-time, column-free miniaturized gas mass spectrometer in detecting target species with partial overlapping spectra are reported. The achievements have been made using both nanoscale holes as a nanofluidic sampling inlet system and a robust statistical technique. Even if the presented physical implementation could be used with gas chromatography columns, the aim of high miniaturization requires investigating its detection performance with no aid. As a study case, in the first experiment, dichloromethane (CH_2_Cl_2_) and cyclohexane (C_6_H_12_) with concentrations in the 6–93 ppm range in single and compound mixtures were used. The nano-orifice column-free approach acquired raw spectra in 60 s with correlation coefficients of 0.525 and 0.578 to the NIST reference database, respectively. Then, we built a calibration dataset on 320 raw spectra of 10 known different blends of these two compounds using partial least square regression (PLSR) for statistical data inference. The model showed a normalized full-scale root-mean-square deviation (NRMSD) accuracy of $10.9\mathrm{\%}$ and $18.4\mathrm{\%}$ for each species, respectively, even in combined mixtures. A second experiment was conducted on mixes containing two other gasses, Xylene and Limonene, acting as interferents. Further 256 spectra were acquired on 8 new mixes, from which two models were developed to predict CH_2_Cl_2_ and C_6_H_12_, obtaining NRMSD values of 6.4% and 13.9%, respectively.

## Introduction

The combination of the most recent results in the micro and nanotechnologies^1^ with specific assessed approaches of analytical gas instruments is changing the way measurements^2^ can be carried out. Novel generations of analytical instruments that use developments in the field of Micro-Electro-Mechanical Systems (MEMS) and Nano Electro-Mechanical Systems (NEMS) open perspectives for devices with a very high level of miniaturization for Gas Chromatography (GC). Recent advances in Gas-Chromatography–Mass Spectrometry (GC–MS) analytical techniques and more targeted technologies, such as Ion Mobility Spectroscopy (IMS), Surface Acoustic Waves–Mass spectrometry (SAW–MS), and Gas Chromatography–Surface Acoustic Waves–Mass spectrometry (GC–SAW), shows a clear trend at reducing the size, the analysis time as well as the costs of installation and deployment. Therefore, stringent vacuum conditions should be satisfied, requiring complex differential vacuum systems, bulky connections, and expensive vacuum pumps. However, it is challenging to eliminate the need for relatively large gas inlet flows for the instruments. Examples of these efforts could be found in several recent publications, where the various gasses were injected with an sccm value in the range of 10–200 sccm^3–8^. To further reduce flows, a reduction of the whole system’s dimensions was studied by several researchers, obtaining the first consequential reduction of the needed inlet throughputs: for example, in 2007, Kim et al.^9^ reported the first integration of a micro GC, where a 4-stage gas micropump was connected to a microcolumn with a length of 25 cm. This system obtained the best vapor separation between 0.2 and 0.3 sccm. More recently, Hsieh and Kim^10^ developed a microcirculatory gas chromatography system and tested it successfully on the separation of different isomers, working at a fixed flow rate of 0.5 sccm. Similar results were reported using a particular technology called Knudsen Pump (KP), based on parallel channels created with nano orifice membranes. On that topic, Qin et al. wrote several papers^11–13^, developing small systems with a flow of 0.4, 0.82, and 0.15 sccm, respectively. In general, nanotechnology devices can drastically change how these measures could be carried out, allowing radical and extremely relevant system dimensional and power supply reductions. A significant improvement in system simplification^14,15^ is possible by using nanometer-scale orifices^16^ as sampling points and smart gas interfaces toward atmospheric pressure.

As long as MS spectra data processing is concerned, in the literature, several papers have applied multivariate techniques (especially PCA) to spectra measured with GC–MS, focusing mainly on classification problems. These works cover several fields, where food is one of the most active: for example, in 2013 Welke et al.^17^ used mass spectrometry detection in conjunction with PCA and Stepwise Linear Discriminant Analysis (SLDA) to discriminate between 5 different types of wine, with a success rate of 100%. Lv et al.^18^ used GC–MS to acquire fingerprint spectra of Puerh green tea and six other green teas and then used Cluster Analysis (CA) and PCA to evaluate the difference between the Puerh variant and the other ones. More recently, Mogollon et al.^19^ performed GC–MS acquisitions on Ecuadorian spirits beverage, whose samples were prepared with a particular technique called Headspace Solid-phase microextraction (HS-SPME). This pretreatment, in conjunction with PCA, allowed them to use mass spectrometry for a valuable quality inspection of these alcoholics. Other studies were conducted on humans: for example, Jha et al.^20^ analyzed human body odor data acquired with GC–MS with Kernel PCA (KPCA). This technique allowed them to find volatile compounds that could act as biomarkers, obtaining a good classification between different subjects. In 2019, Stark et al.^21^ applied more complex deep learning techniques to MS data acquired on melanoma samples, trying to classify them between a melanoma or non-melanoma mole. Three different deep learning algorithms were explored: Single Layer Perceptron, 1-Hidden Layer Multilayer Perceptron, and 5-Hidden Layer Multilayer Perceptron, with the second one giving better results (63.3% of correct classifications). Jajin et al.^22^ considered techniques such as Soft Independent Modelling of Class Analogy (SIMCA) and Orthogonal Partial Least Squares-Discriminant Analysis (OPLS-DA) to discriminate between subjects affected by medullary thyroid cancer and healthy ones, using spectra from GC–MS on plasma samples. Using OPLS-DA, an R^2^ parameter (coefficient of determination, with maximum 1) value of 0.925 was obtained.

This paper aims to demonstrate the effectiveness of the combined approach of nanotechnologies experimentally and signal processing to achieve real-time MS column-free detection of gaseous compounds. More specifically, we first briefly sketch the physical background based on nano-orifices, allowing us to significantly reduce the readout time and power consumption. Then, we test the instrument with two compounds, intentionally selecting species having partially overlapping m/z spectra to act as interferents to each other. Finally, we use multivariate analysis to develop a simple, computationally light predictive model to get a quantitative evaluation of compounds in both single and combined concentrations. Even if the instrument's physical characterization hints and mechanical implementation will be detailed in a forthcoming paper, we consider it essential here to briefly sketch the key features of the underlying physical technique to fully understand the overall system's potentiality.

### Physical enabling approach

In this section, we will show the critical aspects of the technology that enables real-time mass spectra with the only purpose of giving a brief sketch of the underlying physical approach without entering the details. More specifically, we will discuss why the tested technology could save orders of magnitude in power consumption and response time.

In standard MS systems, molecular ion beams can be generated in several ways and through different techniques, for instance, electronic ionization, discharge ion source, photoionization, etc.^16^. Once generated, the ion beams must fly into a mass filter first to be selected (through a single mass filter or with a tandem mass spectrometer), then through a couple of mass filters with a scattering cell down to a detector to measure the intensity of the selected ions. As well known, to reduce severe scattering effects and consequential losses of the ion beam, it is critical to reach a gas regime for the MS system where the mean free path of the ion is comparable with the geometrical dimensions of the analytical system *D*, flying from the ion source to the detector, alias, achieving a Knudsen number $K=\lambda /D>1$*,* where *D* is the dimension of the vessel and $\lambda$ is the mean free path

1 $$\lambda =\frac{k \cdot T}{\sqrt{2} \cdot {\sigma }^{2} \cdot p}$$

where *T* is the temperature (in Kelvin degrees), $\sigma$ is the scattering cross section, *p* is the pressure (in Pascals), and *k* is Boltzmann's constant. Therefore, to reduce MS losses of the ion beam, it is required to reach a gas regime where the mean free path *λ* of charged particles is comparable with the geometrical dimensions of the analytical system D. Thus, ions should fly from the source to the detector, requiring pressures of the standard analytical system (whose length is about tens of cm) in the range of ${10}^{-6}\div {10}^{-7}\mathrm{ mbar}$. In this case, ions collide mainly with the inner chamber walls rather than each other.

To understand the critical features of nanodevice-based MS, we take the continuity equation of a single vessel having an inlet throughput or gas flow/rate Q (in $mbar\cdot L\cdot {s}^{-1}\equiv W$) and outlet effective pumping speed (or volumetric flow rate) $S=dV/dt$ (in $L\cdot {s}^{-1}$) as

2 $$V\cdot dp=Q\cdot dt-S\cdot p\cdot dt\to -V\frac{dp}{dt}=S\cdot p-Q$$

where again, *V* is the volume of the vessel.

It is easy to show that the differential equation could be solved with boundary conditions as an inverse exponential decay of pressure

3 $$p(t)={\left.\frac{Q}{S}\right|}_{\infty }-\left({{\left.\frac{Q}{S}\right|}_{\infty }- p}_{0}\right){e}^{-\frac{S}{V}t}$$

where ${p}_{0}$ is the initial pressure, ${\left(Q/S\right)}_{\infty }$ is the pressure at stationary regime, and $\tau =V/S$ is the time constant of the system. When the system achieves a steady state, we have

4 $$Q=p\cdot S=p\frac{dV}{dt}$$

Also, at the stationary regime, another equation relates pressures across an orifice through the conductance *C* (in $L\cdot {s}^{-1}$)

5 $$Q=C\cdot ({p}_{2}-{p}_{1})$$

where *p*_*2*_ and *p*_*1*_ are the pressures across the orifice.

**Figure 1:**
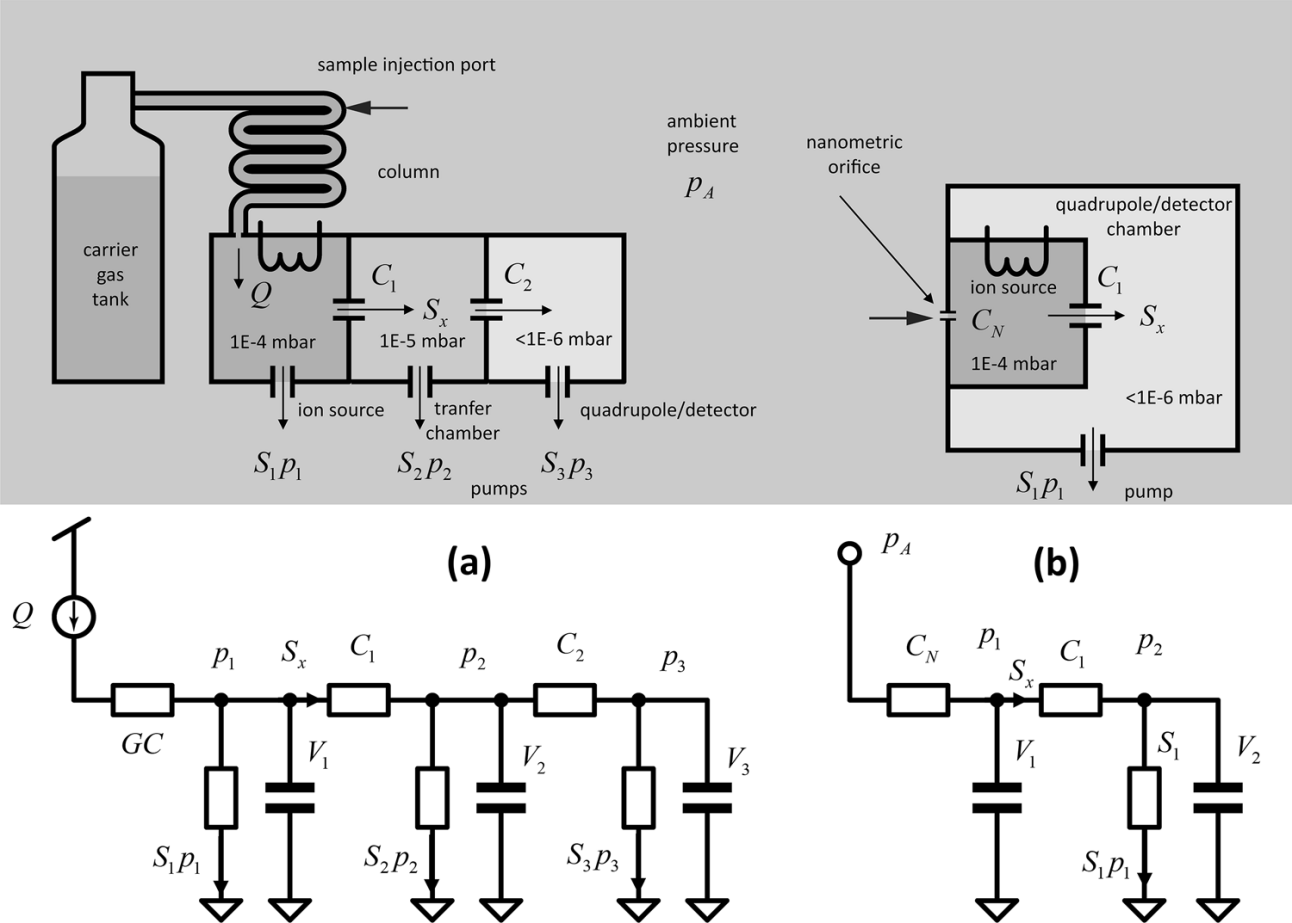
Comparison between conventional approach, (**a**) and nanodevice approach (**b**). The electric model lets us understand the pressure behavior between chambers at transient and steady-state regimes. The model is based on the analogy: throughput—current: $Q\leftrightarrow Sp$, volume – electrical capacitance: $V\leftrightarrow C$, and pressure – electric potential: $p\leftrightarrow V$, since both systems obey to the same differential equations.

When multiple vessels are interconnected by pumps and orifices, the constant mass flow constraint sets, by using (4) and (5) for each vessel, an *N-th* order differential equation that gives pressures at each point under initial conditions. An equivalent electric model is usually defined to better understand the behavior, where electric potential, capacitance, and current are equivalent to pressure, volume, and throughput, respectively, as shown in Fig. 1.

In standard MS systems, the gas sample is eluted into a gas carrier (contained in a tank) to flow into a chromatography column for separation at constant throughput. To achieve molecular regimes, a system of multiple chambers and pumps is used, as shown in Fig. 1A, where $Q={S}_{1}{p}_{1}+{S}_{2}{p}_{2}+{S}_{3}{p}_{3}$ (we used a simplified 3-chamber system to show the concept). As the electrical model shows, we can drop the pressure (electric potential) at constant throughput (electric current). In industrial systems, a standard unit for *Q* is the SCCM@1 bar (in ${cm}^{3}\cdot min\cdot 1bar\equiv 1.66W$), using 10 SCCM@1 bar as a typical value. With this value, it is easy to show that for such a flow and using a unique chamber, the pumping speed would be technically impractical, requiring multiple chambers. However, the power effort is anyway generally relevant. As a rough example, pump speeds of *S*_*1*_ ~ *700 L/s*, *S*_*2*_ ~ *300L/s*, and *S*_*3*_ ~ *300L/s* would require electric power of about *700 W* using turbomolecular and rotary oil vane pumps.

Conversely, using a nanometric orifice by sampling the gas to be analyzed at ambient pressure allows for achieving molecular regimes with a reduced number of chambers and power requirements, as shown in Fig. 1B. Moreover, the dominant time-constant $\tau ={V}_{1}/{S}_{x}$ (since the inter-chamber conductivities are much lower than output effective conductivities) is dramatically decreased. More specifically, in standard systems, we have typical values of *V*_*1*_ ~ *1000 cm*^*3*^ and *S*_*x*_ ~ *0.1–1 L/s*, while for the proposed technique, it could be *V*_*1*_ ~ *1 mm*^*3*^ and *S*_*x*_ ~ *0.1–1 L/s*, thus reducing the time constant of orders of magnitude. Finally, a note should be pointed out about the use of the column. Even if the proposed approach could also be used with GC, which is now easier to miniaturize and implement in MS (as reported recently by Bristow et al.^23^ or by Huang et al.^24^, for example), the possibility of avoiding its use altogether, with a consequent decrease in costs and acquisition time, is worth exploring. In other words, even if the GC could highly increase the selectivity of the overall system by introducing the separation as an additional information variable, it is a focal point of this paper to show a robust identification of compounds without using gas columns.

Using nanodevices allows for a dramatic decrease in the complexity of the overall system using a single or multiple (array) orifices at a nanometric level in molecular flow, achieving very low conductances. In the molecular flow regime, rather than considering the collective motion of the fluid, we can focus on the motion of the single molecule, flying “practically alone” from one end to another of a pipe and only on a statistical base. In this case, a conductance *C* (in $L\cdot {s}^{-1}$) does not depend anymore on the pressure (as in Poiseuille’s equation) at its ends but only on its geometry, average molecule velocity (or temperature), and molecular mass as

6 $$C = \frac{1}{4}\cdot {\left(\frac{8kT}{\pi m}\right)}^\frac{1}{2}\cdot A=\frac{1}{4}\cdot {\left(\frac{8kT}{\pi m}\right)}^\frac{1}{2}\cdot \frac{V}{l}$$

where *A* is the surface of the aperture, *T* (in ^0^* K*) is the temperature of the gas, *V,* and *l* are the nano-orifice’s volume and depth, and *m* is the molecular mass (in *kg*) of the gas analyte. More specifically, using (6) for a 490 nm round hole diameter and 250 nm depth, we get a conductance of about 21.7 nL/s for air particles which means, by (4) a throughput at ambient pressure of about 1.3 × 10^–3^ SCCM@1 bar, thus several orders of magnitude lesser than standard mass spectrometer that is using about 10 SCCM@1 bar. Using a more sophisticated formula^16^ considering 3D second-order effects, we can get a conductance of about 25% lesser; however, the reference of (6) is still valid for a quick check of the model in different experimental conditions. The dependence of the conductivity versus the molecular mass (lighter gases enter at a higher rate because $C$ is higher for the weightier gases) should not be of concern because the same effect occurs at the exit flow. Therefore, we get the same gas concertation sampled at atmospheric pressure but a much lower pressure level under the mass balance equation.

To summarize, the nanometric orifice technique achieves the following advantages towards standard MS systems: (i) simplified mechanical implementation and reduced power consumption; (ii) reduced sampling time-constant; (iii) reduced throughput. These characteristics allow measurements in real-time and drastically simplify the analytical device making it portable for identifying and quantifying compounds in a complex environmental matrix.

Further advantages not covered in this paper are that the flows through conductance in molecular regime do not give origin to gas collective motions preventing condensation’s effects, chemical reactions, and even clogging events^15^. New micro/nano-scale techniques under investigation allow for trimming ionization pressure into the ion source volume by nano-orifice actuation^14^ to maximize the final sensitivity of the analytical instrument^14^, thus minimizing the measurement time and leading to further system simplification.

### Statistical inference and predictive model setup

Spectra are signals that carry a massive amount of information. However, it is widespread over the whole spectra components, making it very difficult to glimpse a trend from raw data. For the above reason, sensor design should maximize the amount of conveyed information^25^ and needs to use a predictive model by signal processing on spectra raw data. The final goal of this work is to create a model able to infer the concentration of CH_2_Cl_2_ and C_6_H_12_ from a spectrum measured by the mass spectrometer. This was obtained thanks to a multivariate statistical analysis called Partial Least Square Regression (PLSR), a modeling strategy introduced by Wold in 1975^26^. PLS is based on the idea that a whole spectrum could be seen as a single point in a *K*-dimensional space, where *K* is the number of the acquired variables (frequency, wavelengths, u/e^−^ etc.). Generally, a group of *N* spectra could be defined as *N* observations described by a series of *K* variables or a cloud of *N* points in a *K*-dimensional space. So, a spectra dataset is arranged in a matrix ***X**** (*$N\times K$*)*, also referred to as a “data matrix” containing *N* spectra, each defined by *K* variables. In addition to the ***X*** dataset, it is considered an output matrix ***Y*** $(N\times M)$ (where *M* is the number of outputs), containing the value of the variable of interest (in our case, the concentrations of chemical substances) linked to every one of the *N* spectra form the ***X***. PLSR identifies new directions in the data space, called latent variables (LVs), which try to maximize at the same time the variance of ***X***, the variance of ***Y,*** and the covariance between the two. Mathematically, this could be summarized by the fact that PLSR can divide the X matrix into two arbitrary matrices, ***T*** $(N\times A)$ (score) and ***W*** $(A\times K)$ (loadings), following the formula

7 $${\varvec{X}}={\varvec{T}}{{\varvec{W}}}^{\boldsymbol{^{\prime}}}+{\varvec{E}}$$

where ***W*** maximizes the variances along the new directions, minimizing in this way the error residual matrix ***E*** $(N\times K)$*.* Moreover, for the assumption stated before, the score matrix T is not only a good predictor of ***X*** but also of ***Y***

8 $${\varvec{Y}}={\varvec{T}}{{\varvec{C}}}^{\boldsymbol{^{\prime}}}+{\varvec{F}}$$

where ***C*** and ***F*** are, respectively, the loadings and the residual matrices of ***Y***. The maximization of variances and covariance is achieved through iterative solutions between (7) and (8). Therefore, at the end of iterations, merging (7) and (8), we have

9 $${\varvec{Y}}={\varvec{T}}{{\varvec{C}}}^{^{\prime}}+{\varvec{F}}={\varvec{X}}{\varvec{W}}{{\varvec{C}}}^{^{\prime}}+{\varvec{F}}={\varvec{X}}{\varvec{B}}+{\varvec{F}}$$

**Figure 2:**
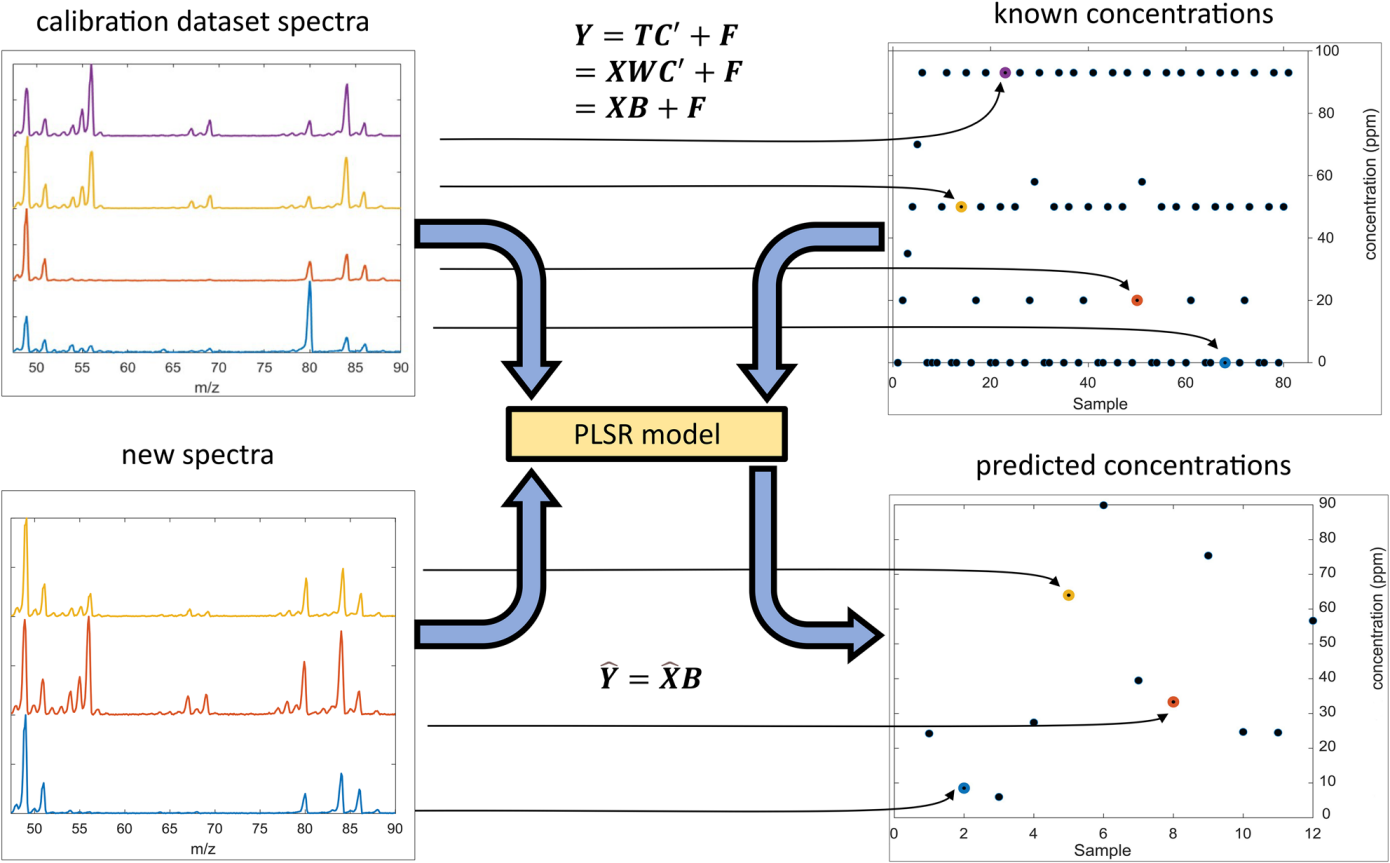
Partial Least Square Regression (PLSR). The ***X*** calibration dataset (upper-left) represents the spectra obtained with the mass spectrometer, each linked with the respective C_6_H_12_ concentration (***Y*** calibration dataset, upper right). The datasets are used as input for creating a PLSR model and calculating an array of calibration coefficients ***B***, allowing us to create the model to infer unknown concentrations $\widehat{{\varvec{Y}}}$ from new spectral inputs $\widehat{{\varvec{X}}}$.

From (3) is easy to see how the PLSR accomplishes the prediction of the variable of interest: the algorithm estimates an array of coefficients ***B*** ($K \times M)$, which allows us to use the linear equation

10 $$\hat{\user2{Y}} = \hat{\user2{X}}\user2{B}$$

to easily predict the value of the variable of interest, $\widehat{{\varvec{Y}}}$ starting from a newly acquired spectrum, $\widehat{X}$**.** In our case, *M* = *2*: that is the concentration of CH_2_Cl_2_ and C_6_H_12_, and $\widehat{{\varvec{X}}}$ could be as small as one spectrum, becoming a $(1\times K)$ matrix. A summary of the whole technique is depicted in Fig. 2. A more detailed description of Principal Component Analysis (PCA), which PLS is based on, can be found in the Supplementary file.

### Materials and experimental setup

**Figure 3:**
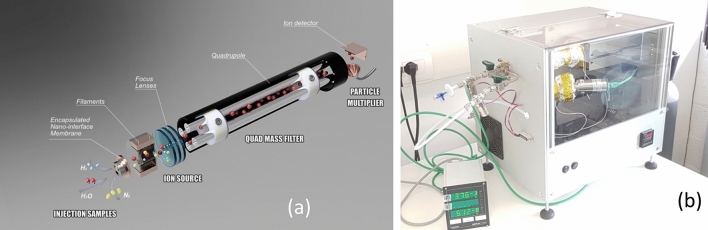
Mechanical conceptual structure of the nano interface integrated into an ion source (**a**) and prototype implementation (**b**). The image was generated by the Fusion 360 software (https://www.autodesk.eu/products/fusion-360/overview?term=1-YEAR&tab=subscription&plc=F360).

**Figure 4:**
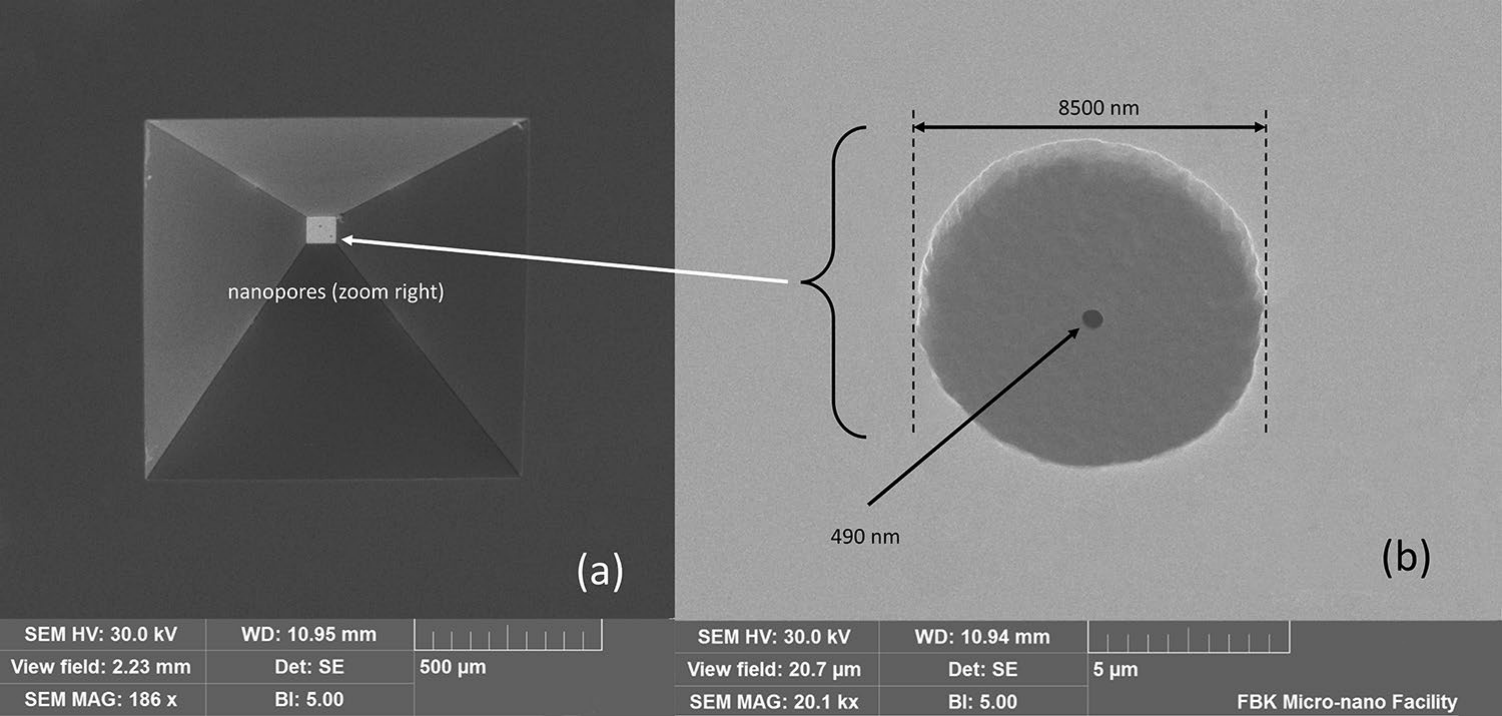
SEM image of a membrane from the chip backside (**a**) with detail of the sub-membrane and orifice (**b**)^2^. SEM image from EVO Zeiss instruments (https://www.zeiss.com/microscopy/en/products/sem-fib-sem/sem/evo.html), courtesy of Fondazione Bruno Kessler (Trento, Italy).

An exploded conceptual view drawing of the miniaturized mass spectrometer is shown in Fig. 3A and is based on a combination of micro and nano technologies (MEMS and NEMS) with techniques currently used for analytical measurements. The analytical prototype is equipped with a nano gas sampling device realized through nanoscale orifices directly interfaced with standard components such as an ion source, ion lenses, mass filter, and a detector to directly sample targets at atmospheric pressure. The experimented prototype is shown in Fig. 3B. An encapsulated nanomembrane interface^2^ uses nanometric orifices and acts as a smart sampling device operating directly at atmospheric pressure in the molecular regime. The sampled inlet gas flows directly into an ion source, where an ion beam is generated. Then, a single quad mass filter can select defined ions detected through a Faraday cup or a second channel electron multiplier (CEM), simplifying the vacuum system and consequently realizing measurements in real-time. The holes' typical diameter is 500 nm, even if this technology allows high versatility around specific needs. Depending on the application, it is possible to realize membranes with single or arrays of orifices tailored to compounds in a complex matrix at reduced concentration^2^. A quadrupole mass filter (CIS 300 by Research Systems) is equipped with a closed ion source and with a second channel electron multiplier (model 4220 Stanford Research Systems) as a detector; the spectrum could be recorded by setting the CEM Voltage to amplify the signal also for less concentrated samples. An SEM microphotograph of the nanometric orifice is shown in Fig. 4 where a membrane-in-membrane structure was adopted, and smaller sub-membranes are realized where nanoscopic holes are created^2^. Several devices were fabricated and tested, having hole diameters from 300 to 600 nm on a membrane side of 80 μm. The device used for the experiments presented in this paper has the following characteristics: a mass range of 1–300 m/z, a typical mass resolution of 0.8 m/z, and a detection limit of around 1 ppm (depending on the compound under analysis).

The purpose of the experiment is twofold: on the one hand, to acquire in real-time MS spectra with the system without the aid of a GC column; on the other hand, to characterize the ability of the system to distinguish between target gases having partially overlapping mass peaks spectra utilizing a predictive model. This study aims to show the potential of the approach to characterizing complex analytical matrices to involve volatile organic compounds (VOCs). Thus, we have chosen two common solvents usually used in analytical applications: dichloromethane (CH_2_Cl_2_ with a molecular weight of 85u) and cyclohexane (C_6_H_12_ with a molecular weight of 84 u), offering a typical case study in terms of complexity and experimental difficulties. More specifically, they are both volatiles showing relatively large fragmentation patterns ranging between 40 and 85 u; there are regions of the spectrum where the fragments generated during the electron ionization process overlap between their relative mass peaks. CH_2_Cl_2_ and C_6_H_12_ compounds were diluted in a gas matrix of argon prepared using three-liter bags and a polypropylene valve with a septum that was used to inject the liquid standards into the bag. Liquid compounds used for sample preparation were obtained from Merck (Darmstadt, DE). All the gaseous solutions were prepared on the day of use and stored in the same environmental conditions of temperature and pressure. For the preparation of the gas samples, a matrix of argon (99,9999%) (Nippon Gases), Tedlar® bags (Restek, PA, US), and a flowmeter (Brooks Scientific, DE) were used to fill bags.

### Samples preparation

For the preparation of Tedlar bags standards, the Full Evaporation Technique (FET) was used^27^. This technique used a small amount of pure sample (a few μL), reducing the operator’s exposure to toxic substances. The FET was based on a transfer of analytes from a condensed matrix, liquid or solid, into a confined vapor phase^27^: the analytes were induced to evaporate into the Tedlar bag until a condition of equilibrium in a short time was reached under the condition *P* < *P*^o^, where *P* is the pressure of the moles of analytes in the volume of the bags at a temperature of work, and P^o^ is the saturated vapor pressure of the sample. The injection tube of the Tedlar® bag was connected to the flowmeter, and the bag was flushed with argon for 18 min at ambient temperature and a primary pressure of 2 bar until 2.4 L using a flowmeter. Then, a small volume of the liquid matrix (in the order of μL) was injected into the septum of the bag using a gas-tight syringe, and they were left to evaporate in the gas matrix to obtain the stock solution. Using a 25 mL gas-tight syringe, the diluted solutions were prepared and injected a few mL of the stock solution was put into the Tedlar® bag filled with 2.4 L of argon. The concentrations were chosen to cover the dynamic range of the instrument: between the detection limit (6 ppm for CH_2_Cl_2_ and 20 ppm for C_6_H_12_) and around one order of magnitude greater to avoid saturation problems with the device. This method was used to prepare 6 ppm, 30 ppm, and 58 ppm solutions for CH_2_Cl_2_, and 20 ppm, 50 ppm, and 93 ppm solutions for C_6_H_12_, respectively. In addition to the bags with only CH_2_Cl_2_ and C_6_H_12_, other bags with both were prepared using the same method. The concentrations of CH_2_Cl_2_ and C_6_H_12_ used for the dataset for a total of 10 mixture combinations are reported in Table 1.

**Table 1:** Concentration of CH_2_Cl_2_ and C_6_H_12_ and their mixtures analyzed in the experiment.

[CH_2_Cl_2_] (ppm)	[C_6_H_12_] (ppm)
6	0
30	0
58	0
0	20
0	50
0	93
30	50
30	93
58	50
58	93

A second acquisition campaign was then performed to create models that could predict the concentration of the two gasses in a more complex mix, where two other gasses, Xylene and Limonene, act as interferents. The sample preparation method remains the same, and 8 new mixes were measured. The concentrations of the 4 gasses in these mixes are reported in Table 2

**Table 2:** Concentration of CH_2_Cl_2_ C_6_H_12_, Xylene and Limonene and their mixtures analyzed in the second experiment.

[CH_2_Cl_2_] (ppm)	[C_6_H_12_] (ppm)	Xylene	Limonene
0	0	0	80
0	0	84	0
6	20	84	80
9	40	84	80
15	75	84	80
30	20	84	80
30	50	84	80
58	50	84	80

### Spectra acquisition

**Figure 5:**
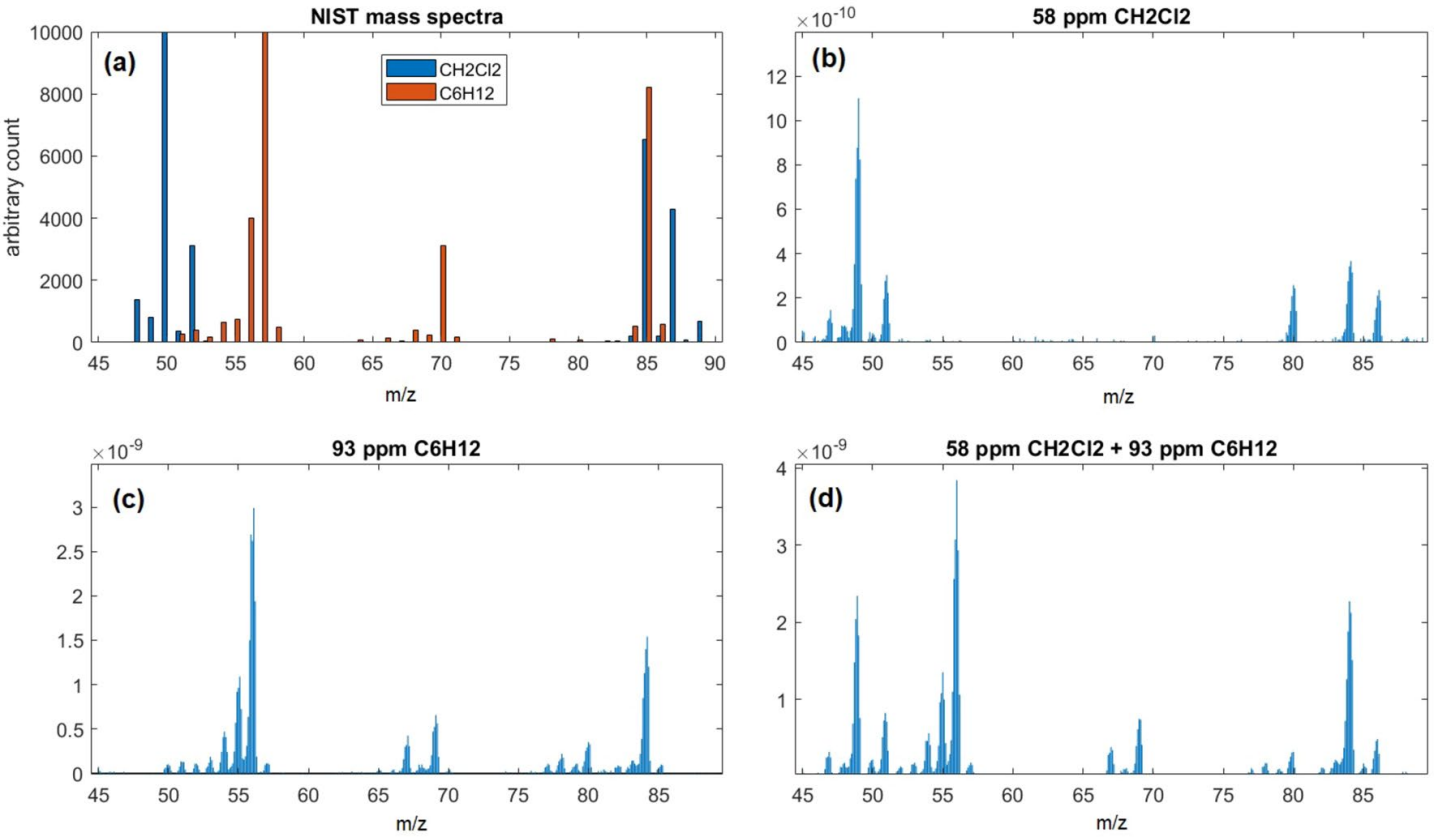
Mass spectra recorded at 45–90 u/e^−^. Each spectrum acquisition lasts about 60 s. Mass spectrum of 58 ppm of CH_2_Cl_2_ (**a**), 93 ppm of C_6_H_12_ (**b**), and 93 ppm of C_6_H_12_ combined with 58 ppm of CH_2_Cl_2_ (**c**). NIST mass spectra (at 70 eV Electron ionization energy) of CH_2_Cl_2_ and C_6_H_12_ (**d**).

The chip membrane hosting nanodevice orifices was positioned between the external environment's high-pressure side (about 1013 mbar) and the low-pressure side toward the quadrupole, allowing to carry out samplings at constant pressure. No chromatographic column upstream was used. The Tedlar bag was connected to the sample holder compartment, and the gaseous sample was flushed a few minutes before recording the mass spectrum. Spectra have been recorded from 45 to 90 u/e^−^, for the concentrations indicated in Table 1 where each spectrum acquisition lasts about 60 s. At first, the samples containing only CH_2_Cl_2_ and C_6_H_12_ were examined. Figure 5B and C show an example of the mass spectra of the analytes in the maximum concentration of the experiment, 58 ppm for CH_2_Cl_2_ and 93 ppm for C_6_H_12_, respectively.

Then, the mass spectra of the gaseous mixtures were recorded, where an example (58 ppm CH_2_Cl_2_ plus 93 ppm of C_6_H_12_) is shown in Fig. 5D. As a reference, the NIST spectra of the two species are shown in Fig. 5A. Due to the absence of the GC that would differentiate the analytical species based on the different retention times, it is apparent how spectra are partially overlapped, and significant fragmentation peaks (the main at 84 u/e^−^) could be seen. To build the calibration dataset, repetitive and automated spectra have been recorded, as detailed in the Methods section.

**Figure 6:**
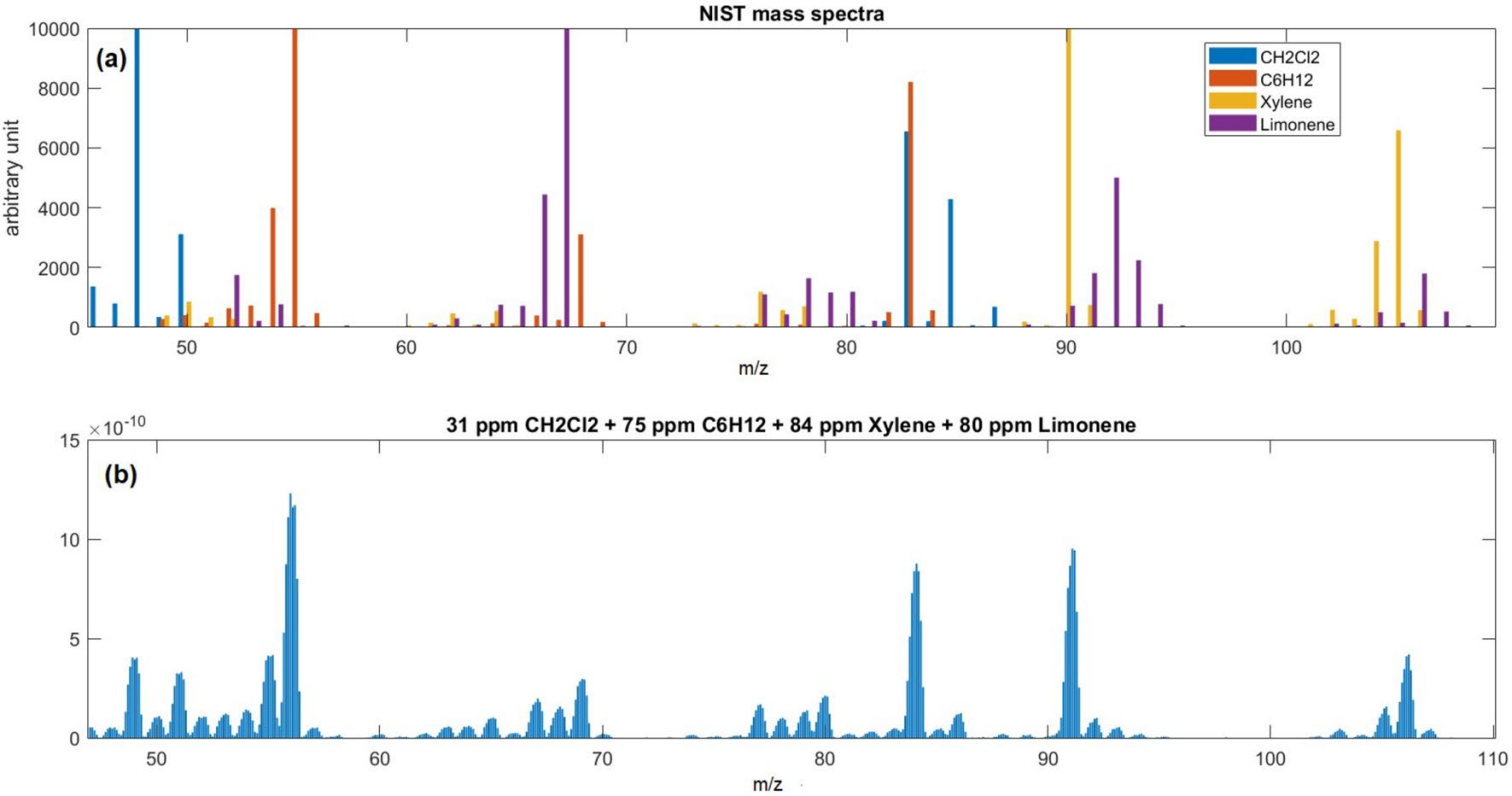
Mass spectra recorded in an interval of 47–110 u/e^−^. NIST mass spectra (at 70 eV Electron ionization energy) of CH_2_Cl_2_, C_6_H_12_, Xylene, and Limonene (**a**). Mass spectrum of a mix of 31 ppm of CH_2_Cl_2_, 75 ppm of C6H12, 84 ppm of Xylene, and 80 ppm of Limonene (**b**).

In a second acquisition campaign, we acquired, in the same way, the mixes presented in Table 2, in the range of 47 to 110 u.m.a. The NIST spectra of the 4 components are shown in Fig. 6A, and an example of the spectra of one of these mixes is depicted in Fig. 6B.

## Methods

### Spectra analysis

**Figure 7:**
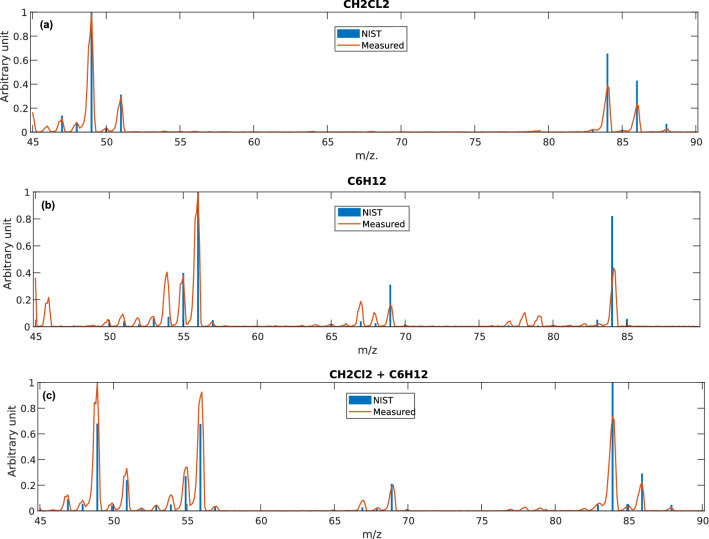
Comparison between acquired spectra and NIST reference for CH_2_Cl_2_ (**a**), C_6_H_12_ (**b**), and CH_2_Cl_2_ + C_6_H_12_ (**c**).

To understand the raw data consistency, we estimated the error between acquired data and NIST reference normalized data^28^. More specifically, it is well known that MS spectra are subject to non-linearities due to physical system and electronic readout, so the global error should be evaluated. Therefore, the spectra have been normalized to the maximum peak for singular and composed compounds, as in NIST references. Then, the data are compared using correlation coefficient^29^ after an alignment pre-processing (see Data augmentation and preprocessing section). A comparison between the mean of the experimental data and the NIST reference is shown in Fig. 7. The correlation coefficient was calculated in three cases: only CH2Cl2, only C6H12, and a mix of both. The r value was calculated for every acquisition (32 for each mix), and then we calculated the mean and the standard deviation. The results are the followings: r = 0.470 ± 0.020 for C_6_H_12_, r = 0.571 ± 0.012 for CH_2_Cl_2_, and r = 0.420 ± 0.010 for the compound C_6_H_12_ + CH_2_Cl_2_. Therefore, it is apparent that spectra acquisition undergoes non-linear effects that alter the ratio between peaks, an essential feature of the spectrum fingerprint. Moreover, the low std values showed us that the acquisition on the same mix was highly repeatable. We will see in the Discussion subsection that notwithstanding the deformation of spectra, the multivariate analysis will be able to overcome the problem, thanks to the construction of a dataset based on known references.

The PLSR model was created using PLS_Toolbox^30^ (Eigenvector Research, inc., WA), which works in a Matlab environment. Among the predictive models to be built on spectra dataset^31–38^ (considering clustering or non-clustering models), we focused on PLSR2^39^ because we aimed at estimating semi-quantitative concentrations of CH_2_Cl_2_ and C_6_H_12_ with a two-variable outputs model (*M* = *2*). We used 12 new spectra as a test set to investigate the model prediction ability, measured on a single gas or a combination of the two. The goodness of the model fit was assessed with a coefficient of determination ($${R}^{2})$$ to measure how well the regression predictions approximate the real data points, with a maximum value of 1.

### Data augmentation and preprocessing

The ***X*** calibration dataset was created with spectra acquired for each one of the CH_2_Cl_2_ and C_6_H_12_ mix reported in Table 1 for a total of 320 acquisitions, collecting about 32 spectra of 427 points for each combination. To reduce noise and concurrently perform data augmentation, we calculated the mean of 20 random spectra for each combination for a total of 100 averaged spectra for a final ***X*** dataset, where *K* = *427* and *N* = *100*. From experimental data, it was found a shift between the spectra peaks due to an intrinsic error of the mass spectrometer around ± 0.25 u/e^−^, so we applied a Matlab function named *icoshift*, developed by Savorani et al.^40^ on averaged spectra. Finally, before being used as input for the PLSR model, spectra underwent a preprocessing called *autoscale*, which consists of the mean centering and scaling of each variable to unit standard deviation.

The same preprocessing was also performed on the spectra acquired during the second campaign, obtaining 80 aligned spectra. For these data, errors due to a new acquisition system were minimized thanks to normalization to the Xylene: as it is possible to see from Fig. 6A, for the u.a. value of 106, the resulting spectrum is influenced only by the amount of Xylene (and by Limonene in a negligible way). So, given the fact that in the acquired mixes reported in Table 2, the Xylene is used in only two concentrations (0 and 84 ppm), for all the 80 spectra (as well as the ones used as a test) the peak value corresponding to the u.a. value of 106 was normalized to 2 references values among all those measured. In particular, we choose the highest value measured for both concentrations: 1*10^–11^ for 0 ppm and 7.25*10^–10^ for 84 ppm. For each spectrum, the ratio between the measured peak in 106 u.a. and the corresponding reference value was calculated, and the whole spectrum was multiplied by that ratio.

## Results

**Figure 8:**
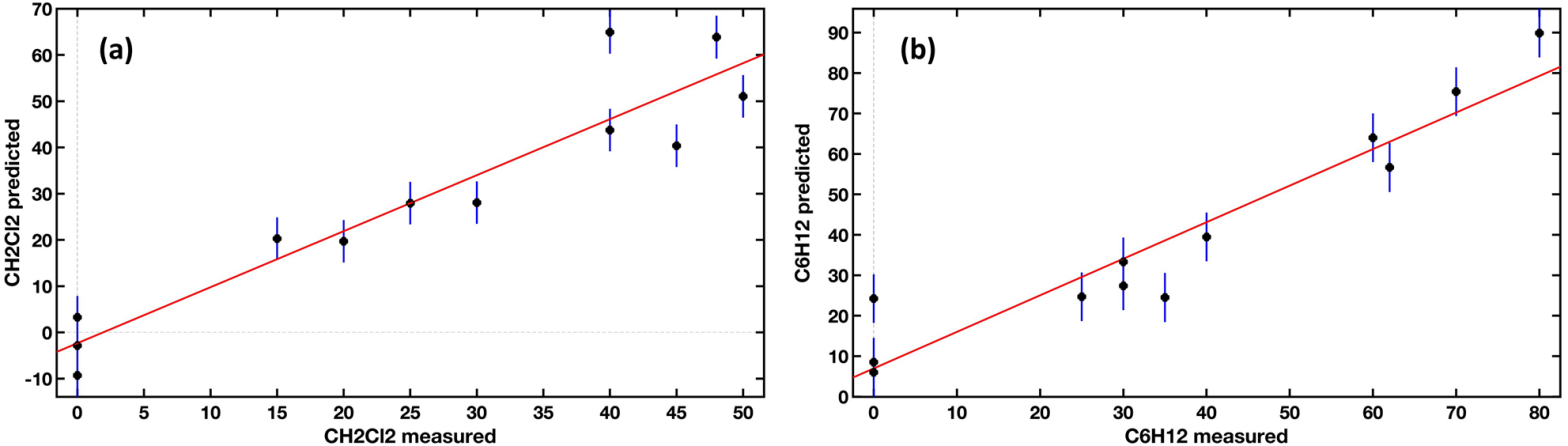
Predicted and measured concentrations with prediction model error bars for CH_2_Cl_2_ (**a**) and C6H12 (**b**). Units in ppm.

Model results show a reduced number of latent variables, LVs = 5, and appreciable values of the coefficient of determination, ${R}^{2}$= 0.886 for CH_2_Cl_2_ and ${R}^{2}$ = 0.900 for C_6_H_12_. The model was tested with spectra measured on 12 unknown mixes of CH_2_Cl_2_ and C_6_H_12_, not present in the ***X*** calibration dataset, averaged, and aligned as described before. The relationship between measured and predicted concentrations is shown in Fig. 8, where prediction model error bars are also displayed. Considering all the numerical values, the estimated concentrations of gasses obtained with the PLSR are quite good: the accuracy is estimated in normalized full-scale root-mean-square deviation (NRMSD) accuracy of $10.9\mathrm{\%}$ for C_6_H_12_ and $18.4\mathrm{\%}$ for CH_2_Cl_2_. Note that the accuracy considers the presence of both species in detection.

**Figure 9:**
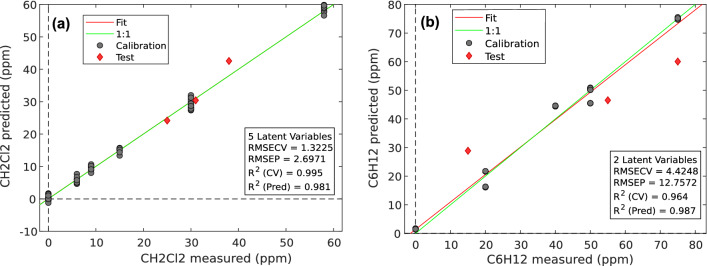
Prediction plots of the two models created with the spectra acquired on mixes of 4 components. (**a**) Prediction of CH_2_Cl_2_ (**b**) Prediction of C_6_H_12_. In both plots, the black dots represent the spectra used to create and calibrate the model, and the red dots are the spectra used as the test set. The green line is the bisector of the plot, representing the ideal prediction, whereas the red line represents the linear fit of the model.

With the data acquired on the second campaign, two models were created, for the prediction of CH_2_CL_2_ and C_6_H_12_, respectively: with the addition of Xylene and Limonene, the differences in the accuracy made us prefer two models that predict a single variable to a single model that predicts both. Given the simplicity of the calculation required for the variable prediction (see Eq. (10)), the variation in the calculation time using two models instead of one is negligible. These two models were tested with 3 unknown mixes containing all 4 elements. The prediction plots are shown in Fig. 9: the black dots represent the spectra used for the creation and the calibration of the models, and the red dots the test spectra. The results were very good: for CH_2_Cl_2_, we obtained an R^2^ of 0.981 and an NRMSD of 4.7%; whereas for C6H12, we obtained an R^2^ of 0.987 and an NRMSD of 17%.

## Conclusions

This paper has shown quantitative multivariate analysis results of experimental spectra from a nanodevice-based mass spectroscopy system where no chromatographic column upstream was used. A data set was constructed on 320 raw spectra derived from 10 different blends of two compounds with overlapped peaks acting as interferents. The model showed an accuracy with an NMRSD error of $10.9\mathrm{\%}$ and $18.4\mathrm{\%}$ for each species, respectively, even in combined mixtures. Then, a second dataset was created with 256 spectra acquired on mixes of the two compounds and another two gasses used as interferents. One model for each of the two compounds was created, and they both showed good prediction ability, with NMRSD of 4.7% and 17%, respectively. The accuracy of the model could be increased by widening the ***X*** calibration dataset with more acquisitions in a higher number of different concentrations for the two gases, using a more extensive and time-consuming setup. However, once the model is built offline, it could be easily implemented in a real-time detection system with very low computational resources (A more detail description of the computational requirements of a model can be found in the supplementary file).

## Supplementary Information

Supplementary Information.

## Data Availability

The datasets used and/or analyzed during the current study are available from the corresponding author upon reasonable request.
