# Supplementary material for: Real-time gas mass spectroscopy by multivariate analysis
Source: Sci Rep. 2023 Apr 13;13:6059. doi: 10.1038/s41598-023-33188-x (PMC10101983; doi:10.1038/s41598-023-33188-x)
Supplement: Supplementary file 1 — Supplementary Information. [file 41598_2023_33188_MOESM1_ESM.pdf]

PCA

PCA finds a subset $A < K$ of directions that still contain most of the total variance of data. From a geometrical point of view, these new directions, called Principal Components (PCs), define an A-dimensional subspace of the original K-dimensional data space. Generally, a data matrix could be decomposed into two arbitrary matrices, $\boldsymbol{T}$ $(N \times A)$ (score) and $\boldsymbol{W}$ $(A \times K)$ (loadings), so as $\boldsymbol{T} = \boldsymbol{X}\boldsymbol{W}'$, where $\boldsymbol{W}$ is an orthonormal matrix. PCA finds $\boldsymbol{W}$ to maximize the variances along the loadings directions. Following classical regression theory, it could be shown that optimal $\boldsymbol{W}$ minimizes the error residual matrix $\boldsymbol{E}$ $(N \times K)$, where

$$\boldsymbol{X} = \boldsymbol{T}\boldsymbol{W}' + \boldsymbol{E}$$

so that we can find the best projection of $\boldsymbol{X}$ (prediction) along loadings directions, in a dot product fashion, as $\widehat{\boldsymbol{X}} = \boldsymbol{T}\boldsymbol{W}'$. It could be shown that loadings are the eigenvectors of the correlation matrix $\boldsymbol{X}'\boldsymbol{X}$. However, the calculation of all eigenvectors would be computationally intensive and subject to experimental noise, especially for lower eigenvalue values. PCA approach reduces to A the number of directions (PCs) along which calculate maximum variance, using numerical iterations (e.g., NIPALS) and uses the residual matrix $\boldsymbol{E}$, containing the difference between the original data matrix $\boldsymbol{X}$ and the new representation in the PCs space, as a control of the convergence to stop iterations. In this way, it is said that $\boldsymbol{T}$ and $\boldsymbol{P}$ are "good summaries" of the original $\boldsymbol{X}$ matrix because, even if it has a reduced dimension, it still contains most of the information

Computational requirements of the model

To assess the portability of the model in a real-time environment, an estimation of the computational requirements is needed. Predictive models often require huge computational requirements (especially convolutional neural networks for image processing) due to 2D convolutions requiring graphical processing units (GPUs). In our case, the multivariate analysis has significantly lesser computational requirements. It is also worth stressing that the use of multivariate analysis is based on two phases, the first of which is performed offline and consists of processing reference datasets to set up the predictive model before the actual measurements, and the second consists in using the predictive model with newly acquired datasets. The overall approach is displayed in Supplementary Fig. To set up the predictive model, multiple spectra with known concentrations are acquired in a database. Then, they are averaged and aligned as described in the Method section and used as input for the predictive model calculated offline by statistical tools. Once the predictive model is completed, we obtain the weight matrix $\boldsymbol{B}$, which is then implemented in the chosen hardware for digital processing. More precisely, for a given acquired input spectrum $\widehat{\boldsymbol{X}}$, a basic output is simply given by $\widehat{\mathbf{Y}} = \widehat{\mathbf{X}}\mathbf{B}$. The computational requirements for the matrix product are the execution of K sums of products, either in fixed- or floating-point notations, resulting in linear complexity (O(K) complexity). In our case we have two outputs (*M = 2*), for spectra of 427 points (*K = 427*), thus the computational requirements are very small. However, to improve the quality of results, $\widehat{\boldsymbol{X}}$ may undergo a run of spectrum alignment with selected reference spectra (see Data augmentation and preprocessing subsection). For this purpose, approaches exploiting correlation shifting of spectral intervals and FFT engines provide reduced computation times, due to their linearithmic complexity with *K*log(K)* complexity[1]. Such linear or linearithmic complexities are very much treatable for real-time analyses even by single-core low-cost CPUs, microcontrollers, or DSPs. As a reference, in our tests, the Python interpreter running on a Raspberry Pi Zero W requires less than 2.6 seconds to process an acquired spectrum of 427 points. If N input spectra are acquired and M output variables are required, computation times for alignment increase by N, and matrix products by $N*M$. Then, with N and M in the order of unity, computation times are still expected in the order of seconds in our reference architecture[2]. However, it is worth noting that matrix math extensions have been available on conventional CPUs since the end of the 20th century[2] and can provide significant speed-ups. To summarize, once the predictive model is set up, the multivariate approach allows for the execution of challenging achievements with relatively low computational cost and on low-cost hardware compared to other machine learning (ML) approaches.

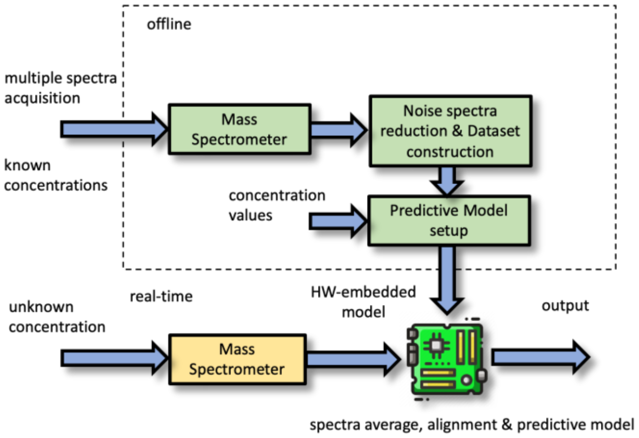

Supplementary Fig.1 Approach of the predictive model setup and implementation.

References

1. Savorani, F., Tomasi, G. & Engelsen, S. B. icoshift: A versatile tool for the rapid alignment of 1D NMR spectra. *J. Magn. Reson.* **202**, 190–202 (2010).
2. Peleg, A. & Weiser, U. MMX technology extension to the Intel architecture. *IEEE Micro* **16**, 42–50 (1996).
